# Regulation of constitutive vascular endothelial growth factor secretion in retinal pigment epithelium/choroid organ cultures: p38, nuclear factor kappaB, and the vascular endothelial growth factor receptor-2/phosphatidylinositol 3 kinase pathway

**Published:** 2013-01-03

**Authors:** Alexa Klettner, Daniel Westhues, Jens Lassen, Sofia Bartsch, Johann Roider

**Affiliations:** 1 University of Kiel, University Medical Center, Department of Ophthalmology, Germany; 2 Helmholtz Institute of Ophthalmology, Sadovaya-Chernogryazskaya, Moskow, Russia

## Abstract

**Purpose:**

The retinal pigment epithelium (RPE) is a major source of vascular endothelial growth factor (VEGF) in the eye. Despite the role of VEGF in ocular pathology, VEGF is an important factor in maintaining the choroid and the RPE. Accordingly, the VEGF is constitutively expressed in RPE. In this study, the regulation of constitutive VEGF expression was investigated in an RPE/choroid organ culture.

**Methods:**

To investigate VEGF regulation, RPE/choroid of porcine origin were used. VEGF content was evaluated with enzyme-linked immunosorbent assay. The influence of several molecular factors was assessed with commercially available inhibitors (SU1498, bisindolylmaleimide, LY294002, nuclear factor kappaB [NFkB] activation inhibitor, mithramycin, YC-1, Stattic, SB203580). For toxicity measurements of inhibitors, primary RPE cells of porcine origin were used, and toxicity was evaluated with methyl thiazolyl tetrazolium assay.

**Results:**

VEGF secretion as measured in the RPE/choroid organ culture was diminished after long-term (48 h) inhibition of vascular endothelial growth factor receptor-2 by VEGFR-2-antagonist SU1498. VEGF secretion was also diminished after phosphatidylinositol 3 kinase was inhibited by LY294002 for 48 h. Coapplication of the substances did not show an additive effect, suggesting that they use the same pathway in an autocrine-positive VEGF regulation loop. Inhibition of protein kinase C by bisindolylmaleimide, on the other hand, did not influence VEGF secretion in organ culture. Inhibition of the transcription factor SP-1 by mithramycin displayed effects after 24 h and 48 h. Inhibiting hypoxia-inducible factor-1 (HIF-1) and Stat3 did not show any influence on constitutive VEGF secretion. Inhibition of the transcription factor NFkB diminished VEGF secretion after 6 h (earliest measured time point) and remained diminished at all measured time points (24 h, 48 h). The same pattern was found when the inhibitor of mitogen-activated kinase p38 was applied. A combination of NFkB and p38 inhibitors displayed an additive effect, completely abolishing VEGF secretion.

**Conclusions:**

Constitutive VEGF secretion in the RPE/choroid seems to be regulated by the transcription factor NFkB and the mitogen-activated kinase p38 in an independent manner. Constitutive VEGF secretion may be regulated to a lesser extent by the transcription factor SP-1, while Stat3 and hypoxia-inducible factor-1 do not seem to be involved. Additionally, VEGF secretion seems to be regulated long-term by an autocrine positive loop via vascular endothelial growth factor receptor-2 and phosphatidylinositol 3 kinase.

## Introduction

Vascular endothelial growth factor (VEGF) is the major physiologic growth factor in angiogenesis in the developing organism [1,2]. In the retina, VEGF is mainly responsible for the development of the retinal vasculature [3]. In the adult organism, VEGF is foremost considered a pathological factor in the development of choroidal neovascularization in age-related macular degeneration (AMD) or of macular edema diabetic retinopathy [4,5], but VEGF has important functions in the healthy adult retina. VEGF is a survival factor for endothelial cells and important for the maintenance of the choroid [6,7]. Additionally, VEGF protects the retinal pigment epithelium (RPE), Müller cells, photoreceptors, and retinal neurons [8-11], and may save axotomized ganglion cells from delayed cell death [12].

VEGF expression and secretion are regulated on many levels by various factors, such as different transcription factors [13,14], protein kinases [15], and receptor signaling [16]. The exact pathways involved in induced VEGF secretion depend on the stimulus, and little is known about the regulation of constitutive VEGF in the eye. For ocular tissue, a differential involvement of mitogen-activated protein kinases (MAPK) has been shown [17], as p38 is involved in constitutive VEGF expression and secretion, while extracellular signal-regulated kinase-1/2 accounts only for oxidative stress–induced VEGF increase, which is likely a transient phenomenon [18]. In addition, for VEGF, autoregulation has been implicated in ocular as well as in other tissue [19-21].

The aim of this study was to characterize the constitutive regulation of VEGF secretion and expression in ocular tissue. We focused on transcription factors, signaling kinases, and autoregulative functions on the constitutive VEGF secretion in an RPE/choroid organ culture.

## Methods

### Perfusion organ culture

Organ culture was prepared as described previously [22]. Briefly, to prepare the RPE/choroid sheets, freshly slaughtered pig eyes were cleaned of adjacent tissue and immersed briefly in antiseptic solution. The anterior part of the eye was removed, the RPE/choroid sheet was separated from the sclera, and prepared tissue was fixed between the lower and upper parts of a fixation ring. Organ sheets were cultivated in a perfusion chamber (Minucells & Minutissue, Bad Abbach, Germany). In this chamber, basal and apical tissue was not separated [22]. The chamber was placed on a heating plate and perfused with medium (Dulbecco’s modified Eagle’s medium (PAA, Cölbe, Germany) and Ham F12 medium (PAA; 1:1) supplemented with penicillin/streptomycin (1%), L-glutamine, HEPES (25 mM), sodium-pyruvate (110 mg/ml), and 10% porcine serum (PAA). The flow rate was 2 ml/h. The gas exchange takes place via the silicone tubes and the pH and CO_2_ content of the media was stabilized with HEPES. The perfusion of the tissue allows a steady-state equilibrium of the tissue [23].

### Treatment of the organ culture

At the second day of cultivation, tissue sheets were exposed to the designated inhibitors (SU1498 [VEGFR-2 inhibitor; Calbiochem, Darmstadt, Germany]: 10 μM; bisindolylmaleimide [PKC inhibitor; Calbiochem]: 1 μM; LY294002 [PI3K inhibitor; Calbiochem]: 25 μM; NFkB inhibitor [Calbiochem]: 1 μM; mithramycin [SP-1 inhibitor; Sigma-Aldrich, Munich, Germany]: 1 μM; Stattic [Stat3 inhibitor; kind gift of Dr. Thorsten Berg]: 1 μM; YC-1 [HIF-1 inhibitor; Calbiochem]: 25 μM; SB203580 [p38 inhibitor; Calbiochem]: 10 μM) or combination of inhibitors (SU1498 + LY294002: 10 μM/25 μM; SU1498 + bisindolylmaleimide: 10 μM/1 μM; SB203580 + NFkB inhibitor: 10 μM/1 μM; SB203580 + SU1498: 10 μM/10 μM; SU1498 + NFkB inhibitor: 10 μM/1 μM), and the experiment was conducted as described elsewhere with modifications [17]. Briefly, medium was collected for 1 h before treatment (time point 0 h). After collection, perfusion of the tissue was interrupted, and the medium was transferred to a Falcon tube (BD Bioscience, Heidelberg, Germany) where the respective inhibitor was added to the medium. The medium was transferred back into the chamber, incubated for 20 min, and the perfusion was restarted. Additionally, the inhibitor was added to the medium reservoir. For untreated cultures, the same procedure was conducted without any addition of a substance. The supernatant was collected at designated time points (6 h, 24 h, 48 h) for 1 h, centrifuged for 5 min at 16,200 ×g and stored at −20° C until further evaluation.

### Calcein stain

After 48 h of treatment with the respective inhibitor, the tissue was incubated with calcein AM (AnaSpec, Inc., San Jose, CA) for 30 min, washed with Dulbecco’s PBS (PAA), and the RPE cells were observed using a fluorescence microscope, with λex/λem=497/517 nm (Zeiss, Jena, Germany). Calcein AM is widely used as a membrane permeability marker that readily passes through the cell membrane of living cells. After non-fluorescent calcein AM permeates the cytoplasm, it is hydrolyzed by endogenous esterase into the highly green fluorescent calcein, which is retained in the cytoplasm. Therefore, calcein AM can be used to distinguish live and dead cells through the cytoplasm green fluorescence [22].

### Evaluation of vascular endothelial growth factor content

The VEGF content was measured with a VEGF-enzyme-linked immunosorbent assay (ELISA; R&D Systems, Wiesbaden, Germany) following the manufacturer’s instructions. The range of detection of the ELISA was between 15 pg/ml and 1046 pg/ml. The ELISA detects all isoforms of VEGF-A and readily detects porcine VEGF-A [19]. The data are depicted as % of VEGF amount at time point 0 h.

### Retinal pigment epithelium isolation and cell culture

RPE cells were isolated as previously described [19]. Briefly, freshly slaughtered pig eyes were cleaned of adjacent tissue and immersed briefly in antiseptic solution. The anterior part of the eye was removed, as well as the lens, vitreous, and retina. In each eyecup, trypsin (PAA) was added, and incubated for 5 min at 37° C. The trypsin solution was removed and substituted with trypsin-EDTA for 45 min at 37° C. RPE cells were removed from the choroid by gently washing them of the choroid with the trypsin-EDTA (PAA) used for incubation, collected in media, and washed. Cells were cultivated in Dulbecco’s modified Eagle’s medium supplemented with penicillin/streptomycin (1%), L-glutamine, amphotericin B (0.5 μg/ml), HEPES (25 mM), sodium-pyruvate (110 mg/ml), and 10% fetal calf serum (Linaris GmbH, Wertheim-Bettingen, Germany).

### Treatment of cells

Confluent cell cultures of passage 3 were treated with the designated inhibitors or a combination of inhibitors at the same concentrations used in the organ culture treatments (see above) and incubated for 24 h or 48 h.

### Methyl thiazolyl tetrazolium assay

Twenty-four or 48 h after the designated treatment, cell viability was tested with methyl thiazolyl tetrazolium (MTT, Sigma Aldrich, Munich, Germany) assay as described elsewhere [24]. Briefly, the culture media were discarded, and the cells were washed three times with PBS and incubated for 2 h with 0.5 mg/ml MTT in Dulbecco’s modified Eagle’s medium at 37 °C. After incubation, the MTT solution was discarded, and dimethylsulfoxid (DMSO; Roth, Karlsruhe, Germany) was added to the cells. Cells were shaken at 200 rpm for 5 min on an orbital shaker, the DMSO was collected, and the absorbance was measured at 555 nm wavelength. Untreated control was defined as 100% survival.

### Statistics

Each experiment was independently repeated at least three to five times. Significant changes were calculated with an unpaired Student *t* test for organ culture and MTT assay. A p value less than 0.05 was considered significant. The bar charts depict the mean and standard deviation of the three to five experiments. In the bar charts, significance is depicted as follows: + p≤0.05, ++ p≤0.01, and +++ p < 0.001.

## Results

### Toxicity of the inhibitors

**Figure 1:**
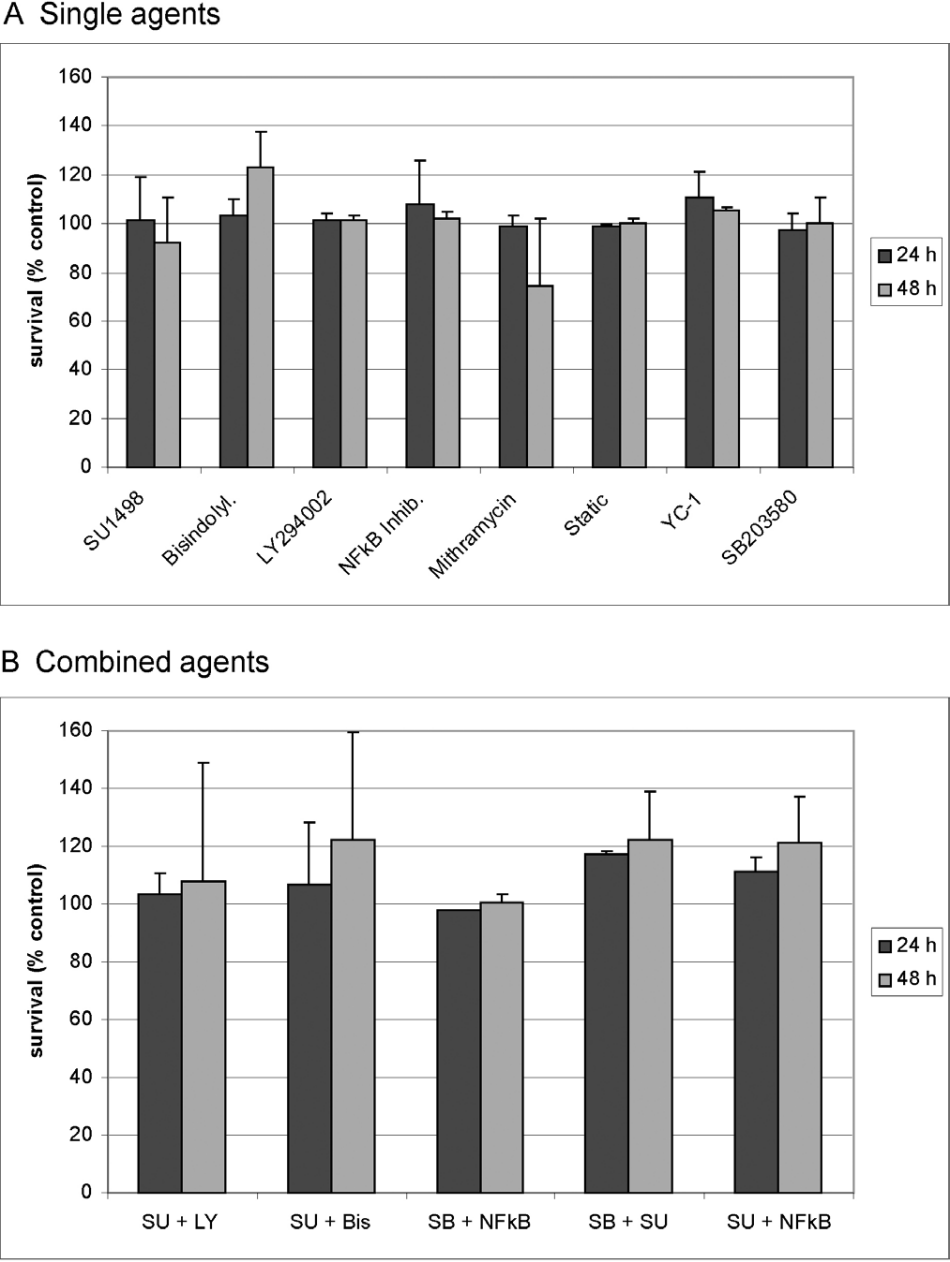
Toxicity of applied biochemical agents. Primary retinal pigment epithelium cells were treated with (**A**) indicated inhibitors and (**B**) a combination of inhibitors for 24 h or 48 h and assessed for cell viability in an MTT assay. Untreated cells served as control. None of the inhibitors displayed a significant toxicity applied concentrations: SU1498: 10 μM; Bisindolylmaleimide: 1 μM; LY294002: 25 μM; NFkB inhibitor: 1 μM; Mithramycin 1 μM; Stattic 1 μM; YC-1: 25 μM; SB203580: 10 μM. Abbreviations: SU=SU1498; LY=LY294002; Bis: bisindolylmaleimide; NFkB: NFkB inhibitor; SB=SB203580. The bars depict the mean and standard deviation of three to five independent experiments. Statistical significance was determined with the Student *t* test.

None of the applied inhibitors exhibited a significant decline in viability in RPE cell culture at the applied concentrations, either as a single compound or in combination (Figure 1). However, although not statistically significant due to standard deviation, treatment with mithramycin resulted in a decline of cell viability at 48 h.

### Viability of the cultures

**Figure 2:**
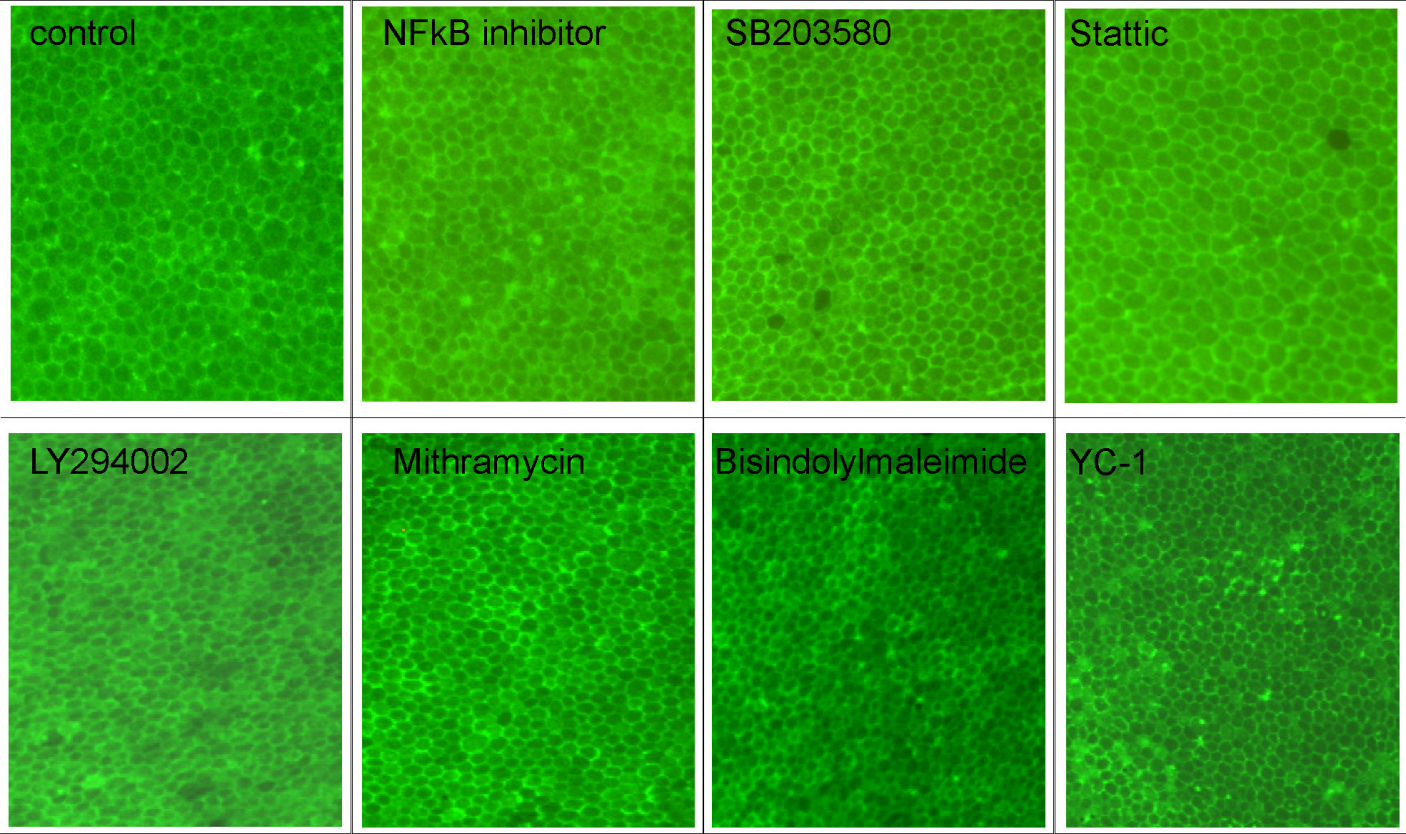
Viability of organ culture. Retinal pigment epithelium (RPE)/choroid perfusion organ cultures were treated for 48 h with indicated inhibitor and stained with calcein. None of the tested inhibitors displayed an effect on RPE viability in the applied concentration. A representative picture of a calcein stain for each inhibitor is shown.

None of the applied inhibitors compromised the viability of the organ culture regarding the RPE cell viability as seen in the calcein stains (Figure 2).

### Vascular endothelial growth factor secretion of organ cultures

The amount of VEGF secreted in the perfusion organ culture differed between the different preparation and donor animals. The mean of the VEGF concentration secreted in 1 h of all perfusion cultures at hour 0 (before treatment) was 242.15 pg/ml, the standard deviation was 152.99 pg/ml, the median was 214.54 pg/ml, and the range over all experiments was 32.53–849.962 pg/ml. The results of the inhibitors are depicted as % of the 0 h control (before respective treatment). The absolute VEGF concentrations at 0 h are given for each set of experiments in the legends for all inhibitors, combinations, and respective controls.

### Influence on vascular endothelial growth factor secretion

Vascular endothelial growth factor receptor-2 pathway (vascular endothelial growth factor-2, phosphatidylinositol 3 kinase, protein kinase C)

**Figure 3:**
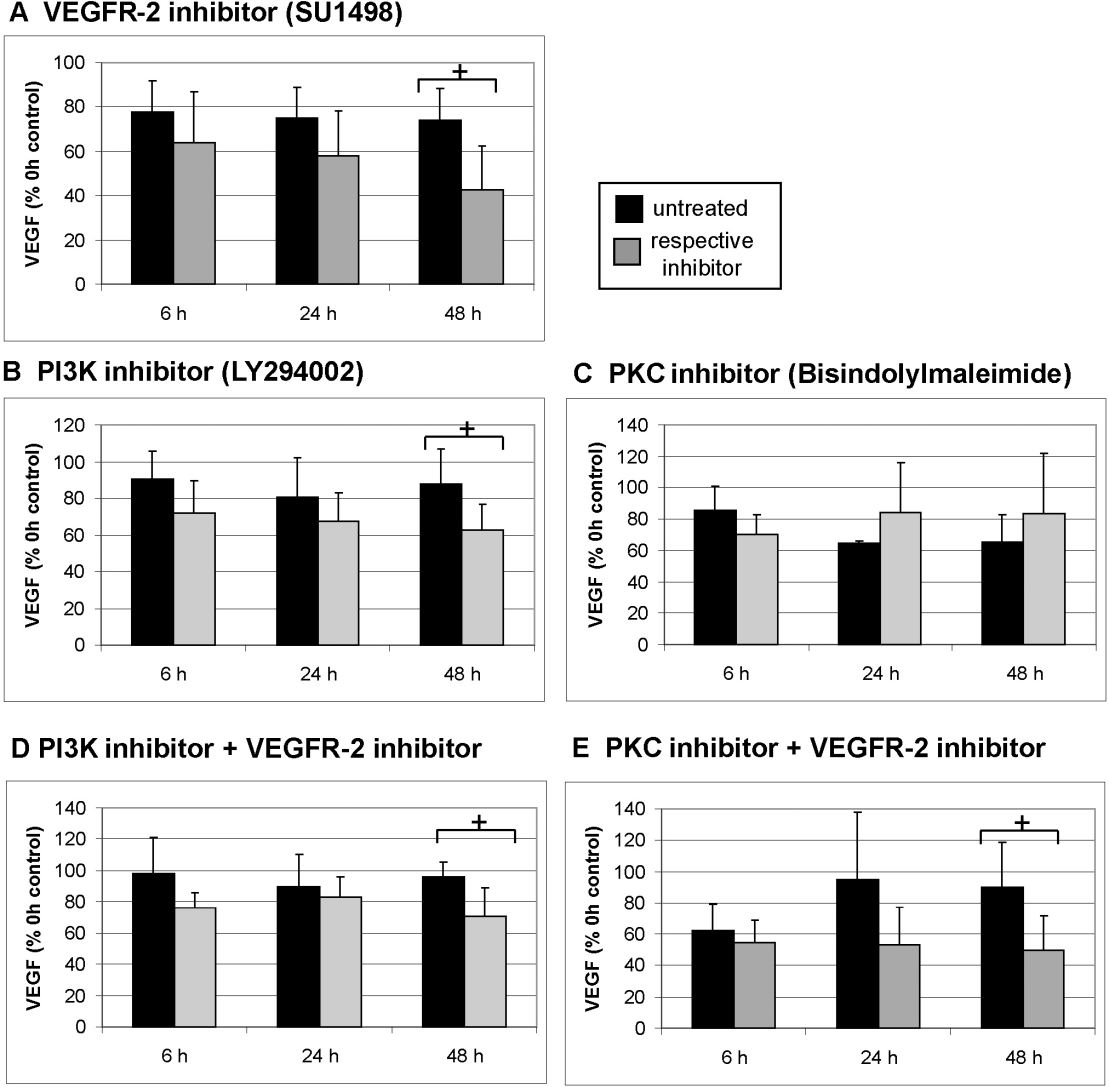
Vascular endothelial growth factor secretion regulation by vascular endothelial growth factor receptor-2 and downstream kinases. Organ cultures at day 2 of preparation were treated with the indicated inhibitors and supernatant was collected for 1 h after 6 h, 24 h, and 48 h. Untreated cultures served as controls. Vascular endothelial growth factor (VEGF) content was evaluated with enzyme-linked immunosorbent assay. The vascular endothelial growth factor receptor-2 (VEGFR-2) inhibitor SU1498 significantly reduced VEGF secretion after 48 h (**A**), so did the phosphatidylinositol 3 kinase (PI3K) inhibitor LY294002 (**B**). Inhibiting protein kinase C (PKC) with bisindolylmaleimide displayed no effect (**C**). The combination of either LY294002 or bisindolylmaleimide with SU1498 mimicked the effect of SU1498 alone (**D**, **E**). The results are depicted as % VEGF at 0 h (before treatment). The bars depict the mean and standard deviation of three to five independent experiments. Absolute concentration of VEGF at 0 h was 301.46±136.17 pg/ml for SU1498, 475.46±208.25 pg/ml for bisindolylmaleimide, and 473.690 ±262.03 pg/ml for LY294002. The absolute concentration of VEGF of the respective controls at 0 h was 390.69±238.69 pg/ml for SU1498, 410.78 +/− 134.4 for bisindolylmaleimide, and 418.30±109.30 pg/ml for LY294002. Absolute concentration of VEGF at 0 h was 307.95±145.19 for the combination of SU1498 and bisindolylmaleimide and 346.91±104.65 for the combination of SU1498 and LY294002. The absolute concentrations for the respective controls were 269.37±149.19 pg/ml for SU1498 and bisindolylmaleimide and 240.3±60.94 for SU1498 and LY29002. Statistical significance was determined with the Student *t* test. Significant is depicted as follows: +p<0.05; ++ p<0.01. +++ p<0.001.

VEGFR-2 receptor signaling was inhibited using VEGFR-2 receptor tyrosine kinase inhibitor SU1498. Exposure of the organ culture to SU1498 for 48 h, but not for 24 h or 6 h, significantly reduced VEGF secretion compared to untreated organ culture at respective time points (p<0.05; Figure 3A). Inhibition of PI3K using LY294002 displayed a similar pattern to what was observed with VEGFR-2 inhibition with a significant reduction in VEGF secretion after 48 h, but not at the other time points tested (p<0.05; Figure 3B). When the substances were applied together, no additional decrease in secretion was observed, indicating a common pathway (Figure 3D). Inhibition of PKC using bisindolylmaleimide did not display any reduction in VEGF secretion (Figure 3C). Coapplication of VEGFR-2 and the PKC inhibitor mirrored the effect of the VEGFR-2 alone (Figure 3E).

#### Transcription factors (hypoxia-inducible factor-1, Stat3, SP-1, nuclear factor kappaB)

**Figure 4:**
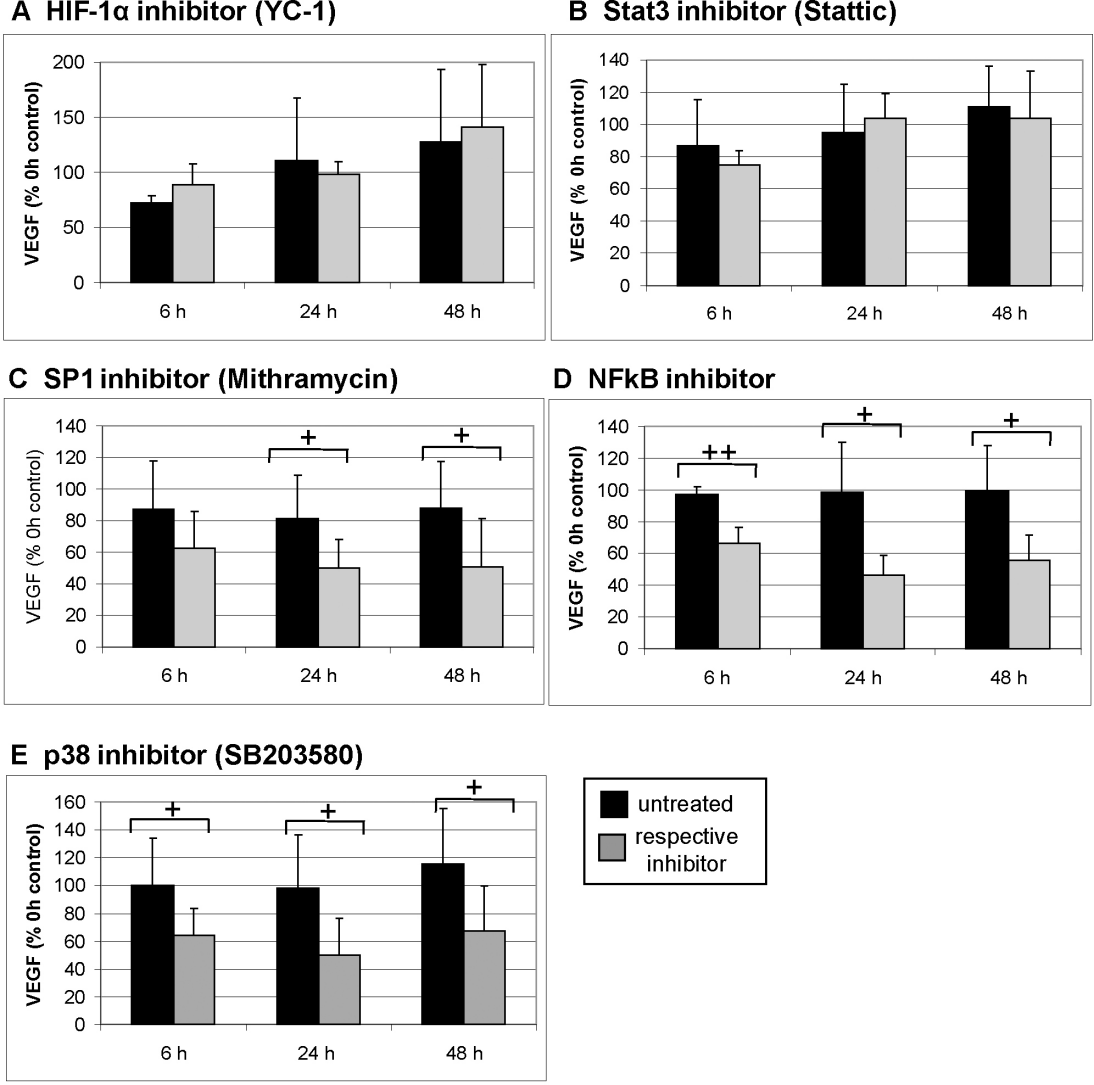
Vascular endothelial growth factor secretion, transcription factors, and p38. Organ cultures at day 2 of preparation were treated with indicated inhibitors, and supernatant was collected for 1 h after 6 h, 24 h, and 48 h. Untreated cultures served as controls. Vascular endothelial growth factor (VEGF) content was evaluated with enzyme-linked immunosorbent assay. Inhibition of HIF-1α with YC-1 or of Stat3 with Stattic displayed no significant effect on VEGF secretion (**A**, **B**). The SP-1 inhibitor mithramycin significantly reduced VEGF secretion after 24 h and 48 h (**C**). The inhibition of nuclear factor kappaB (NFkB) and of p38 with SB203580 significantly reduced VEGF secretion at all time points tested (**D**, **E**). The results are depicted as % VEGF at 0 h (before treatment). The bars depict the mean and standard deviation of three to five independent experiments. Absolute concentration of VEGF at 0 h was 202.03±59.04 pg/ml for mithramycin, 202.68±69.16 pg/ml for the NFkB inhibitor, 235.51±52.31 pg/ml for Stattic, 186.23±13.46 pg/ml for YC-1, and 148.58± 93.84 pg/ml for SB203580. The absolute concentration of VEGF of the respective controls at 0 h was 245.73±78.28 pg/ml for mithramycin, 243.27±22.77 pg/ml for the NFkB inhibitor, 158.43±139.4 pg/ml for YC-1, and 113.90±42.64 pg/ml for p38. Statistical significance was determined with the Student *t* test. Significant is depicted as follows: + p<0.05; ++ p<0.01. +++ p<0.001.

The inhibition of HIF-1 alpha using the inhibitor YC-1 and Stat3, using the inhibitor Stattic, did not display an effect on VEGF secretion (Figure 4A,B). The inhibition of SP-1 using mithramycin significantly reduced VEGF secretion after 24 h and 48 h (both p<0.05; Figure 4C); however, possible toxic effects must be considered (Figure 1A). The inhibition of NFkB using a specific NFkB inhibitor displayed a profound effect on VEGF secretion at all time points tested, which significantly diminished VEGF secretion (6 h: p<0.01, 24 h: p<0.05, and 48 h: p<0.05; Figure 4D).

#### p38

We have previously shown that the MAPK p38 is involved in constitutive VEGF secretion, which was evaluated after 6 h of incubation [17]. In this study, we found that inhibition of p38 using SB203580 significantly inhibited VEGF secretion after 6 h as well as after 24 h and 48 h of incubation (all p<0.05; Figure 4E).

#### Combined agents

**Figure 5:**
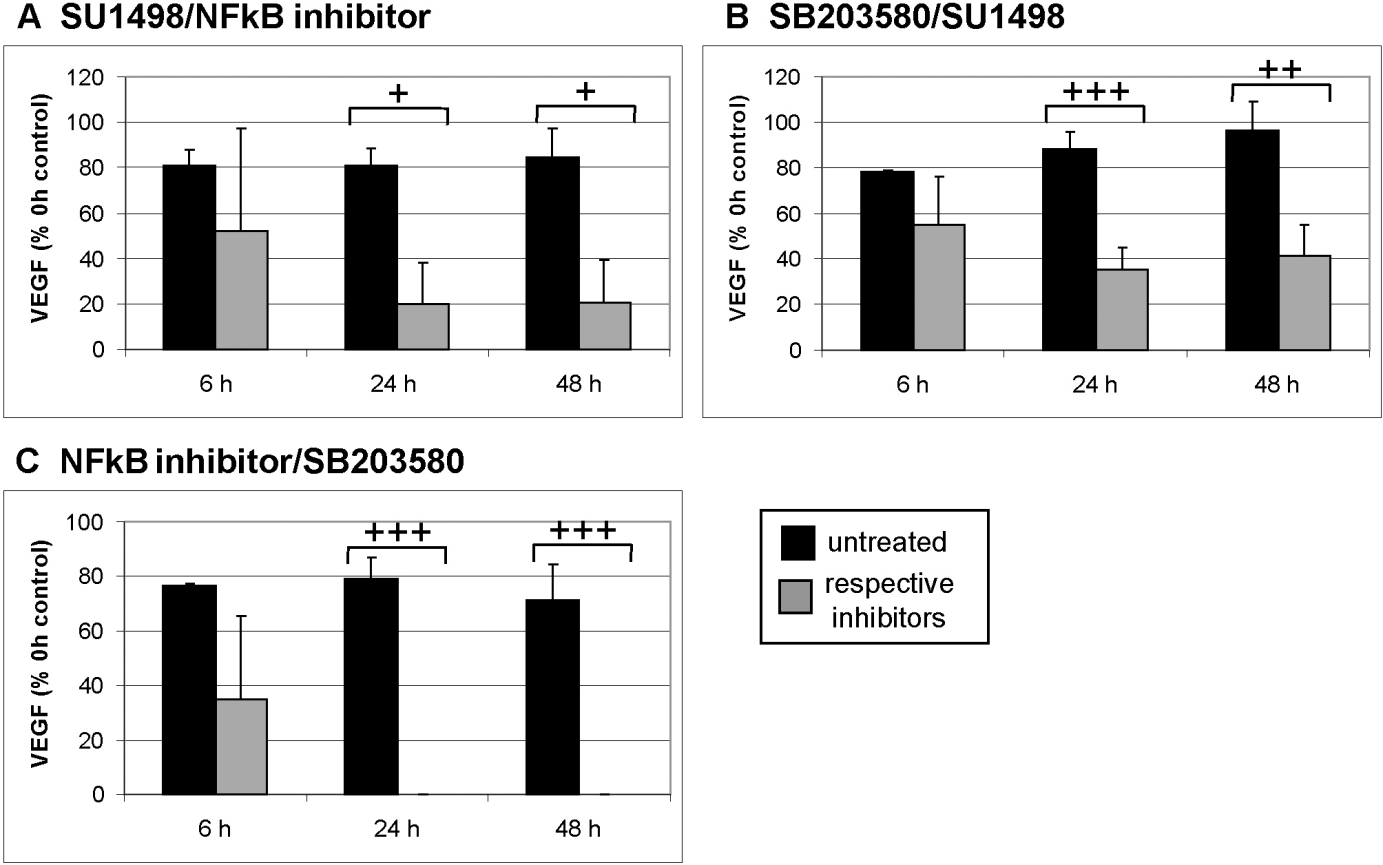
Vascular endothelial growth factor secretion and combinations of nuclear factor kappaB, VEGFR-2, and p38. Organ cultures at day 2 of preparation were treated with a combination of indicated inhibitors and supernatant was collected for 1 h after 6 h, 24 h, and 48 h. Vascular endothelial growth factor (VEGF) content was evaluated with enzyme-linked immunosorbent assay. A combination of SU1498 with either nuclear factor kappaB (NFkB) inhibitor (**A**) or SB203580 (**B**) displayed similar results as the inhibition of NFkB or SB203580 alone. However, a combination of NFkB inhibitor and SB203580 abolishes VEGF secretion, indicating an additive effect of these two inhibitors (**C**). The results are depicted as % VEGF at 0 h (before treatment). The bars depict the mean and standard deviation of three to five independent experiments. Absolute concentration of VEGF at 0 h was 67.08 ±24.08 pg/ml for SB203580 + the NFkB inhibitor, 119.97±50.98 pg/ml for SB203580 + SU1498, and 99.15±41.66 pg/ml for SU1498 and NFkB inhibitor. The absolute concentration of VEGF of the respective controls at 0 h was 82.89±26.90 for SB203580 + NFkB inhibitor, 115.02 ±76.57 pg/ml for SB203580 + SU1498, and 177.23±85.04 pg/ml for SU1498 + NFkB inhibitor. Statistical significance was determined with the Student *t* test. + p<0.05; ++ p<0.01. +++ p<0.001.

NFkB inhibitor combined with SU1498: Inhibition of NFkB with an NFkB inhibitor and VEGFR-2 with SU1498 displayed a similar pattern seen with NFkB inhibition alone (Figure 5A). SB203580 combined with SU1498: Inhibition of p38 with SB203580 and VEGFR-2 with SU1498 displayed a similar pattern seen with SB203580 alone (Figure 5B).

NFkB inhibitor combined with SB203580: When the inhibitors of NFkB and p38 were combined, no VEGF was detected in the supernatant after 24 h or 48 h. This effect is much more profound than the reduction obtained with either SB203580 alone or NFkB inhibitor alone. This indicates strongly that both factors, NFkB and p38, regulate VEGF independently. Furthermore, inhibition of both factors completely abolishes VEGF secretion in this model (Figure 5C).

## Discussion

The expression of the growth factor VEGF is regulated via many factors, including external factors, signal transduction molecules, and transcription factors [25]. Although VEGF can be induced by a wide variety of stimuli, it is constitutively expressed in the RPE, and little is known about the intracellular factors regulating constitutive VEGF expression. In this study, we investigated the regulation of constitutive VEGF secretion in a perfusion organ culture model using biochemical inhibitors. The organ culture model we use has been characterized and shown to be a valuable model for investigations of VEGF secretion, stably secreting VEGF from day 2 to day 5 of cultivation [22], and has been used before to compare the efficacy of VEGF antagonists [19]. Biochemical inhibitors were used, as small interfering RNA or specific antibodies are not feasible or affordable in this system. These biochemical inhibitors have been well described in the literature [26-33]. However, the specificity of some biochemical inhibitors is under debate [34-37], which has to be regarded a limitation of this study.

In our study, we focused on two different aspects of regulation, one being possible autocrine/paracrine VEGF regulation via VEGFR-2, the other investigating different transcription factors that have been associated with VEGF regulation before [15]. Additionally, we further investigated the effect of the MAPK p38 [17].

Possible autocrine regulation of VEGF expression has been suggested before, as the inhibition of VEGF by bevacizumab and ranibizumab reduced the VEGF protein content of RPE cells, measured after 6 h of stimulation [19]. Additionally, in endothelial cells, the external supply of VEGF induced the expression of HIF-1α, an important regulator of VEGF in hypoxia [20]; and in glioblastoma cells, VEGF induced its own expression via VEGFR-2 [21]. In our study, we found that the inhibition of VEGFR-2 by SU1498 reduced VEGF secretion after 48 h of incubation, indicating a long-term effect of autocrine or paracrine regulation. The downregulation of VEGF by tyrosine kinase inhibitors has been suggested for sorafenib and pazopanib [38,39]. Even though these authors’ results conflict somewhat, our results suggest that the inhibiting effect of these multiple tyrosine kinase inhibitors may be transmitted via VEGFR-2. This autoregulatory effect via VEGFR-2 may offer a possible explanation why VEGF inhibitors, which bind to VEGF extracellularly, can reduce intracellular VEGF expression as seen in retinal neurons in monkeys [40], and may offer an additional pathway of VEGF inhibition by VEGF antagonists. Two important pathways of VEGFR-2 signaling are PI3K and PKC. Indeed, the effect of PI3K inhibition resembled the inhibition of VEGFR-2, which was not found for inhibition of PKC. The combination of a VEGFR-2 inhibitor with either of the PI3K or PKC inhibitors mimicked the effect of VEGFR-2 inhibition alone, indicating that this regulation is conducted via a common pathway. The VEGFR-2/PI3K pathway has been shown to control the protection of the RPE against oxidative stress [8], so this pathway may be generally important for conducting regulatory functions in the RPE. However, as our organ culture model does include the choroid, paracrine regulation including the endothelial cells of the choroid may also be possible. Further studies will have to elucidate the exact interplay between the cell types.

The inhibition of the transcription factor Stat3 and HIF-1α did not have an effect on VEGF secretion in our system. Although Stat3 is an important angiogenic factor in cancer and has been shown to be a direct transcriptional activator of VEGF [41,42], the involvement of Stat3 in VEGF regulation in the RPE has not been shown so far, and cannot be implicated by our data. HIF-1α induces the upregulation of VEGF in hypoxia, which has also been shown for the RPE [13,43], but our data imply that HIF-1α is not involved in constitutive VEGF expression within the limitations of this model.

The use of mithramycin, an inhibitor of SP-1, however, resulted in a reduction of VEGF secretion after 24 and 48 h of incubation. SP-1 has been shown to be involved in VEGF gene regulation [13], and our data indicate that SP-1 might play an important role in constitutive VEGF expression in the RPE. As mithramycin might induce toxic effects after long duration incubation, however, the effect seen after 48 h should be considered with care.

The inhibition of the transcription factor NFkB by a biochemical inhibitor exhibited a strong effect on VEGF secretion at all time points tested, 6 h, 24 h and 48 h, suggesting the constant influence of NFkB on constitutive VEGF secretion. NFkB regulates VEGF in different cell types [44,45] and is involved in VEGF upregulation after different stimuli [16,46]. Our data indicate that NFkB may have an important role in maintaining constitutive VEGF secretion in the RPE/choroid complex. NFkB is an important regulator of the innate immune response [47], an inhibitor of autophagic processes [48], and involved in aging [49]; these factors are associated with the development of AMD [50]. Regulation of VEGF expression may yet be another risk factor for the development of AMD regulated by NFkB, making it an interesting target for the prevention of AMD development.

We have previously shown that p38 is involved in regulating constitutive VEGF expression and secretion, shown after 6 h of p38 inhibition [17]. In our current study, we confirmed our earlier findings and showed that this effect is also seen after 24 and 48 h, thus stressing the importance for p38 in constitutive VEGF regulation. The pattern of VEGF reduction in p38 inhibition resembles the pattern displayed by NFkB inhibition. However, these two effects seem to be additive, indicating independent pathways. Moreover, we were able to completely abolish VEGF secretion in our model system by combining these two agents. This drastic effect is usually seen only when extracellular VEGF inhibitors such as bevacizumab or ranibizumab are used [19] and might offer an interesting alternative for VEGF inhibition, and a possible opportunity to fine tune the amount of VEGF available in the retina. Of course, in addition to the limits of using biochemical inhibitors, findings of an in vitro model, moreover from a non-human origin, have to be regarded with caution. The porcine model, however, is a valuable tool for studying possible pharmacological agents, as it is anatomically and genetically much closer to the human situation than rodent models [51]. Additionally, organ cultures reflect the complex in vivo situation more closely than cell culture models. This complexity includes various possible sources of VEGF in this culture system. Generally, the main source of VEGF of the posterior part of the eye is considered the RPE [52]. As the perfusion organ culture is by its nature a tissue with several different cell types, other cells of the choroid may contribute to the VEGF secretion found in the supernatant. Among these cells are melanocytes, macrophages, fibroblasts, endothelial cells, pericytes, and smooth muscle cell [53]. As normal uveal melanocytes do not secrete VEGF [54], contribution by melanocytes to VEGF secretion in our model is not likely. Resident macrophages can be found in the choroid [55]. Although macrophages can be activated to display a proangiogenic phenotype, resident ocular macrophages without additional stimuli generally display an antiangiogenic phenotype [56,57], and are not likely to contribute to the VEGF secretion of the RPE/choroid explants. Choroidal fibroblasts, however, have been shown to express low amounts of VEGF [58,59] and may contribute to the overall VEGF content of our system. Additionally, choroidal endothelial cells in culture express low amounts of VEGF mRNA, which can be enhanced by various stimuli [26,60-63]. Vascular smooth muscle cells in general are involved in angiogenesis and are able to express VEGF, which has been associated with the development of arteriosclerosis [64,65], yet the VEGF expression in the choroid has not been described so far. Retinal pericytes produce VEGF to stabilize and promote the survival of endothelial cells, and this production can be increased by advanced glycation products (AGEs) or transforming growth factor-β [66-68]. For choroidal pericytes, however, this has not been explicitly shown. With these possible contributions of these cell types in mind, the expression of VEGF in these cell types has been mainly shown in cell culture experiments, focusing on upregulation of VEGF under several stimuli. In the eye, RPE has been shown to be the major, if not the only, source of VEGF in the RPE/choroid in the mouse or monkey eye with little or no VEGF expression found in the choroid of an unchallenged eye [52,69-71].

In conclusion, the data presented in our study indicate that VEGF in the RPE/choroid may be independently regulated by p38 and NFkB. Additionally, long-term autocrine/paracrine regulation of VEGF via the VEGFR-2/PI3K is indicated.
